# The heart of social pain: examining resting blood pressure and neural sensitivity to exclusion

**DOI:** 10.1093/scan/nsaf025

**Published:** 2025-03-31

**Authors:** Sarah J Dembling, Nicole M Abaya, Peter J Gianaros, Tristen K Inagaki

**Affiliations:** Department of Psychology, San Diego State University, San Diego, CA 92182, United States; SDSU-UCSD Joint Doctoral Program in Clinical Psychology, San Diego, CA 92182, United States; Department of Psychology, San Diego State University, San Diego, CA 92182, United States; Department of Psychology, University of Pittsburgh, Pittsburgh, PA 15260, United States; Department of Psychology, San Diego State University, San Diego, CA 92182, United States; SDSU-UCSD Joint Doctoral Program in Clinical Psychology, San Diego, CA 92182, United States

**Keywords:** BP-related hypoalgesia, BP-related social algesia, emotional dampening, social rejection, resting blood pressure

## Abstract

Previous work suggests blood pressure (BP) relates to social algesia, where those with higher BP are more tolerant of social pain. The neural correlates of this association, however, are unknown. Based on findings suggesting neural regions involved in physical pain are activated during social pain, the current study explores whether BP relates to subjective and neural responses to social pain, apart from emotional responding. BP was measured, after which participants completed emotional processing and social exclusion functional magnetic resonance imaging (fMRI) paradigms. Results replicated previous findings, with higher systolic BP related to lower trait sensitivity to social pain. However, there were no associations between BP and reported sensitivity to social pain during social exclusion. Moreover, after accounting for adiposity, we found no association between BP and anterior insula (AI) or dorsal anterior cingulate cortex (dACC) activity to exclusion. Finally, there were no reliable associations between BP and reported valence or arousal, or AI and dACC activity to emotional images. Findings partly replicate and extend prior findings on BP and emotional responding to social pain; however, they appear inconsistent with predictions at the neural level. Future experimental manipulation of BP may allow for causal inferences and adjudication of conceptual perspectives on cardiovascular contributions to social algesia.

## Introduction

Social pain—“the adverse subjective experience evoked by actual or potential damage to one’s sense of social connection or social value”*—*is a ubiquitous human experience with many citing social pain experiences as the most negative event in their lives (Jaremka et al. 2011, Eisenberger 2015). For this reason, evolutionary explanations for the relevance of social pain liken experiences of social pain to those of physical pain, underscoring the importance of being accepted in a group and consequences of social disconnection for survival (Eisenberger 2012). That is, just as physical pain alerts us to wounds or ailments that can then be addressed, social pain may alert us of impaired social connection, serving as a “warning sign” for social health. Examples of social pain that may threaten one’s social connections or social value and inflict emotional distress include exclusion from a social gathering, betrayal from a loved one, or undergoing a breakup. Although experiences of social pain are common, how and to what extent individuals experience it differs. Correlates and possible determinants of individual differences in sensitivity to social pain may include aspects of cardiovascular physiology. In particular, findings suggest a link between resting blood pressure (BP) and sensitivity to social pain, with higher BP relating to lower sensitivity to social pain (Inagaki et al. 2018, Umeda et al. 2021, Inagaki and Gianaros 2022). However, the literature has yet to address if individuals’ BP predicts their sensitivity to social pain separately from general emotional responding. Additionally, no studies have examined how a possible shared neural mechanism between BP and social pain—specifically, activity in the anterior insula (AI) and dorsal anterior cingulate cortex (dACC)—may influence the BP–social pain link. Therefore, to extend prior suggestive self-report findings (Inagaki and Gianaros 2022), this study examined the extent to which BP is associated with both self-reported sensitivity and neural activity to social pain, apart from general emotional responding.

### BP and physical pain

There is a well-established literature demonstrating a seemingly counterintuitive finding: higher BP is associated with lower sensitivity to physical pain. That is, increased systolic BP is associated with increased tolerance to pain and decreased subjective ratings of pain (i.e. decreased sensitivity to physical pain; Ghione 1996). This phenomenon, called “BP-related hypoalgesia,” appears in both animal and human studies, across the lifespan, at different levels of hypertension (i.e. in both hypertensive and normotensive individuals) and remains when controlling for covariates known to relate to BP and sensitivity to physical pain (Bruehl and Chung, 2004, Makovac et al. 2020). BP-related hypoalgesia may be mediated by divergent and convergent processes across the cardiovascular and central nervous systems. For example, administration of an opioid antagonist (with no known effects on BP) blocks the link in normotensive male adults (McCubbin and Bruehl 1994). Additionally, changes in BP that induce baroreceptor activation and blunt cortical arousal through interoceptive pathways in male rats result in decreased reactivity to painful stimuli, suggesting increased BP causes decreased pain sensitivity (Dworkin et al. 1979). These findings on possible BP contributions to physical pain have led to questions of whether BP is similarly related to other experiences of pain, such as emotional or social pain.

### BP and social pain

Consistent with social–physical pain theories and BP-related hypoalgesia relationships, recent findings suggest that higher BP does indeed relate to blunted sensitivity to social pain (BP-related social algesia; Inagaki and Gianaros 2024). In one study, higher BP was associated with lower sensitivity to imagined experiences of social pain (Inagaki and Gianaros 2022). Furthermore, other studies have shown that higher systolic BP is associated with lower sensitivity in response to a social exclusion task, Cyberball, in a sample of young adults (Inagaki and Gianaros 2022) and that cardiac systole (when BP is elevated) lessens Cyberball-evoked social pain relative to diastole (Izaki et al. 2024).

Despite these findings, it remains unclear whether BP relates to all emotional experiences or whether there might be some specificity for social pain. Previous theoretical perspectives, particularly the emotional dampening hypothesis (McCubbin et al. 2011), suggest there is a shared mechanism between BP and general emotional responding, in which higher BP is associated with dampened responses to emotional stimuli. Specifically, normotensive individuals with higher BP exhibit lower sensitivity to viewing positive and negative emotional images (Pury et al. 2004, McCubbin et al. 2014). Elevated BP has also been associated with reduced perception of information with affective content (McCubbin et al. 2011). However, in another study, individuals were shown brief video clips of emotional facial reactions to physical pain following the collection of BP (Inagaki and Gianaros 2022). Contrary to findings linking higher BP with lower sensitivity to social pain and previous emotional dampening findings, there was no relationship between BP and sensitivity to emotional responding in this study.

As suggested by these findings, it is possible that BP dampens the most salient emotional responses (Inagaki and Gianaros 2022). To the extent that social pain experiences are the most salient emotional events humans experience, BP would thus predict social pain over emotional responding. Specificity for social pain, however, remains an open question as no studies have presented social pain and general emotional stimuli together within subjects.

### Neural mechanisms in BP and sensitivity to social pain

Another open question in regard to BP-related social algesia is how the relationship may occur. In particular, BP and socio-emotional responding may be explained by the overlapping of neural regions controlling BP and those involved in emotion. Meta-analyses and systematic reviews of the literature suggest that emotional experiences of social pain increase activity in regions overlapping with brain areas thought to be encompassed by networks that have been termed the central autonomic network (Koban et al. 2021) or the allostatic–interoceptive network (Zhang et al. 2023), which regulate and represent BP. These include areas such as the dACC and the AI (Critchley 2005, Wager et al. 2013, Vijayakumar et al. 2017, Wang et al. 2017, Gianaros and Jennings 2018, Mwilambwe-Tshilobo and Spreng 2021). Furthermore, evidence suggests there are shared neural mechanisms between physical and social pain (Rainville 2002, Eisenberger et al. 2006, Dewall et al. 2010, Eisenberger 2012). Specifically, studies have shown increased activity in the dACC and AI during acute experiences of social rejection (Eisenberger et al. 2003, Vijayakumar et al. 2017), social evaluation (Eisenberger et al. 2011, Muscatell et al. 2016), and imaginal re-experiencing of social pain (Kross et al. 2011, Meyer et al. 2015) compared to control conditions. Additionally, reports of greater feelings of social pain correlate with greater AI and dACC activity (Eisenberger et al. 2007, Kross et al. 2011). Both regions reliably activate to experimental manipulations of acute physical pain (Peyron et al. 2000) and are also implicated in meta-analytic reviews of the brain’s involvement in emotional responding (Lindquist et al. 2012). However, these findings exist alongside findings identifying multivariate pattern analysis (MVPA) patterns that demonstrate separate representations of physical pain and social rejection in the brain (Woo et al. 2014). Thus, although some evidence suggests a common central mechanism between BP, social pain, and emotional responding, there is not a consensus on the matter.

At present, no studies have examined the possible neurobiological correlates of the association between BP and sensitivity to social pain or BP and emotional responding. Therefore, we aimed to explore these associations to begin teasing apart social pain from more general emotional responding. To do so, we examine relationships between individuals’ BP and sensitivity to a common form of social pain, namely exclusion, as well as those between BP and sensitivity to emotional images. In particular, we assess whether BP is associated with both self-reported sensitivity to social pain and neural activity to social pain in the AI and dACC. We hypothesize that an individual’s BP predicts their sensitivity to social pain but not emotional responding.

## Materials and methods

### Participants

Participants were recruited from the San Diego community via flyers and social media posts. Our goal was to obtain a dataset of at least 40 individuals with usable data. Thus, 45 individuals were run to guard against data loss due to motion artifacts, attrition, and technical errors. To be included in the study, participants had to be 18 years old or older and fluent in English. Exclusion criteria included identifying as a cigarette smoker, having a mental and/or physical illness diagnosis, actively using psychoactive drugs, being pregnant or planning to become pregnant in the next 6  months, and reporting MRI scanning contraindications. One participant was excluded from all analyses for identifying as a cigarette smoker in the post-scan questionnaire. Therefore, analyses were based on 44 participants (*M*_age_= 23.36, SD = 4.51, range = 18–39 years, 71.1% female). For the neuroimaging analyses only, an additional participant was excluded for excessive motion. Neuroimaging findings are thus based on 43 participants. Informed consent was obtained from all participants and procedures were run in accordance with the San Diego State University Institutional Review Board. Participants were compensated $60 USD for their participation.

### Procedure and measures

Three days prior to the study, participants were sent pre-study instructions to ensure a high-quality BP measure. For at least 2 h prior to the visit, participants were asked to refrain from drinking caffeinated beverages and eating. For at least 24 h prior to the visit, they were asked not to exercise, drink alcohol, or take over-the-counter medications. Participants were also asked to wear short sleeves for accurate BP readings.

Experimental sessions were run between 9 a.m. and 2 p.m. to control for time-of-day variability in BP. Upon arrival, participants reported compliance with pre-study instructions and used the restroom as a full bladder would affect BP readings. Height and weight measures were obtained for body mass index (BMI) calculation, a known correlate of BP (Hubert et al. 1983). Participants then completed the BP measure.

Next, participants completed a resting state scan (reported separately), an emotional images task, and the Cyberball task in the MRI scanner. Following the scan, participants completed the post-scan and trait measures.

### Resting BP measurement

BP was measured using the CARESCAPE V100 Vital Signs Monitor, an oscillometric device. Participants were seated on a chair in a room alone and a BP cuff was placed over the brachial artery of the nondominant arm, positioned at heart level. Participants were instructed to keep their legs uncrossed, feet on the floor, and arms rested on the chair’s armrests. They were left to acclimate to the room for 10 min. Next, a BP measure was taken every 3 min with a total of four measures. Following recommended best practices, an average of the four readings comprised the BP measure (Shapiro et al. 1996). BP was, on average, in the normotensive range with values spanning through stage 2 hypertension (*M*_SBP_ = 106.167, SD = 10.30, range = 85.50–134.75; *M*_DBP_ = 66.81, SD = 8.95, range = 54.50–93.25).

### Emotional images task

To assess neural responding to general emotional content, pictures from the International Affective Picture System (Lang et al. 2008) were presented in a block design that has been previously used for examining responses to emotional images (McRae et al. 2010; Gianaros et al. 2014). Participants were told that they would be viewing a set of images and providing ratings in response to them. Participants saw a total of 48 images: 16 neutral, 16 positive, and 16 negative images. No more than three negative images were shown consecutively. After viewing each image, participants rated valence (1 = “completely unhappy” to 7 = “completely happy”) in response to the question “How happy or unhappy did this image make you feel?” and arousal (1 = “completely calm” to 7 = “completely excited”) in response to “How calm or excited did this image make you feel?” Each image was displayed for 5 s, each question was displayed for 4 s, and a variable (1–3 s) rest period displayed a cross hair between each trial. The image order was randomly generated. There were two variations of the task, each beginning with a neutral image. E-prime software was used to administer the task and record behavioral responses. To input ratings, participants used the scanner’s button box.

### Cyberball

We aimed to examine neural reactivity to an acute experience of social pain elicited by exclusion. Thus, participants completed Cyberball, a virtual ball-tossing game widely used for research on social pain experiences such as ostracism, exclusion, or rejection (Williams and Jarvis 2006). The participant was told that they would play Cyberball, equivalent to “catch” over the internet, with two other players at another university in the area (with names matched to the gender of the participant).

The participant and the two other players were depicted as cartoon images that take turns tossing the ball around to each other. The first two rounds of the game were inclusion rounds, wherein the participant and the other two players passed the ball around to each other. The final round of the game was the exclusion round, wherein once the ball was received by one of the other players, they refrained from passing the ball back to the participant, excluding the participant from the game. The participant pressed “1” or “3” on the MRI-compatible button box to throw to the player on the left or right.

Following previous scanner iterations of the task, participants also completed a shape-matching condition to control for decision-making and pressing buttons. Participants saw a screen with three shapes: one at the top-left, one at the top-right, and one in the bottom-middle. The participant was instructed to match the bottom-middle shape to whichever of the top shapes was the same, pressing “1” to match the bottom shape to the top-left shape, and “3” for the top-right shape.

Once Cyberball was completed, the experimenter told the participant they would thank the other players and then removed the participant from the MRI scanner. Next, the participant was were asked to filled out the Need-Threat Scale (NTS; van Beest and Williams 2006) to assess reported sensitivity to social pain during the exclusion round of Cyberball. Using a 1 = “not at all” to 5 = “extremely” scale, participants indicated “the extent to which [they] felt the following feelings during the last round of the Cyberball (ball-throwing) game.” Sample items included feeling disconnected, rejected, and liked (reverse-coded). Higher scores indicate greater sensitivity to social pain (*α *= 0.879, *M* = 3.352, SD = 0.762, range = 1.333–4.500). After this, the participant was debriefed on the Cyberball deception.

### Trait measures of sensitivity to social pain

To examine how individuals’ general experience of social pain may relate to their BP and to replicate previous findings (Inagaki et al. 2018, Umeda et al. 2021, Inagaki and Gianaros 2022), we collected trait measures of sensitivity to social pain. Trait measures were assessed using Mehrabian’s Sensitivity to Rejection Scale (MSR; Mehrabian 1970), Brief Fear of Negative Evaluation Scale (BFNE; Leary 1983), and Hurt Feelings Scale (HFS; Leary and Springer 2001) following the multivariate functional magnetic resonance imaging (fMRI) scan. A “social pain sensitivity index” was created by standardizing all responses to the same scale (converting the raw scores to *z* scores) and averaging the items to create a single value per participant (α = 0.823). Higher values on the scales indicate greater sensitivity to social pain.

### MRI data acquisition

Subjects were scanned using a 3T Siemens Magnetom Prisma with a 30-channel head coil for a scan time of ∼45 min. Functional images in response to emotional images and Cyberball were acquired using an echo-planar imaging gradient-echo sequence (2.5 × 2.5 × 2.5 mm voxels, repetition time (TR) = 1000 ms, echo time (TE) = 30 ms, 2.5 mm slice thickness, field of view (FOV) = 24 cm, matrix = 96 × 96, flip angle = 59°⁠; simultaneous multi-slice = 4). A resting state scan was also collected but is not discussed here. A T2-weighted structural image was acquired coplanar with the functional images (0.9 × 0.9 × 0.9 mm voxels, TR = 2300 ms, TE = 2.32 ms, 0.9 mm slice thickness, FOV = 24 cm, matrix = 256 × 256, flip angle = 8°⁠).

### Data analysis

#### MRI data preprocessing

MRI data were preprocessed using the fMRIPrep pipeline. For standard language describing methods, see Supplementary material.

#### First- and second-level modeling

Following preprocessing, first- and second-level models were defined. For Cyberball, separate inclusion blocks ranging from 41 to 56 s, a single exclusion block ranging from 69 to 98 s, and three shape-matching blocks (two lasting 36 s and one lasting 40 s) were each modeled separately. Primary comparisons of interest were social exclusion versus inclusion and versus shape-matching control. For the emotional images task, separate neutral, positive, and negative image-viewing blocks (5 s each) and ratings blocks (4 s each) were modeled separately. Primary comparisons of interest were negative versus neutral images and positive versus neutral images. Random effects analyses of the groups were computed using the contrast images generated for each participant.

#### Region-of-interest definition

Based on previous findings and following the preregistration plan, we expected BP to be associated with dACC and AI activity to exclusion (versus inclusion or shape-matching control). Therefore, analyses were constrained to activity within these two regions. Structural regions of interest (ROIs) were defined using the Automated Anatomical Labeling Atlas. To further refine the dACC ROI, we constrained the region at 32 < *y* < 0 on the basis of summary data on cingulate activations to physical pain (Vogt et al. 2003). For the AI ROI, we divided the insula at *y* = 8 the approximate boundary between the dysgranular and granular sectors. MarsBaR (Brett et al. 2002) was used to pull parameter estimates for each condition (exclusion, inclusion, and shape-matching control; negative, positive, and neutral images).

#### BP and sensitivity to social pain and general emotional responding

To test primary hypotheses, Pearson’s correlations were run to assess associations between BP and responses to the NTS (i.e. reported sensitivity to Cyberball), and dACC and AI activity to Cyberball and Emotional Images in Stata (v. 13.1). Associations with systolic and diastolic BP were run separately.

Following the preregistration plan, significant associations with BP were run again with hierarchical linear regressions to assess the strength of the association after adjusting for BMI. Indeed, BP was positively correlated with BMI (*r*_SBP_ = 0.587, *P* < .001; *r*_DBP_ = 0.465, *P* = .002). Significance was determined based on a *P*-value of .05, one-tailed, based on directional hypotheses and following preregistration. Bayes factors (BFs) were also computed in JASP (JASP Team 2024, Version 0.18.3) to better interpret results that did not reach statistical significance with frequentist statistics. BF_10_ values indicate evidence in favor of the alternative hypothesis, whereas BF_01_ values provide evidence in favor of the null hypothesis (e.g. no association between BP and general emotional responding). BFs >3 indicate support for hypotheses, values <0.33 indicate support for null hypotheses, and values between 0.33 and 3 indicate data insensitivity.

Finally, we assessed whether dACC and AI activity to Cyberball account for associations between BP and reported sensitivity to social pain. Mediation models were run using the PROCESS macro for mediation analysis with parameter estimates from a mask of the dACC and AI to exclusion (versus inclusion or versus shape-matching control) as the mediator between BP and sensitivity to social pain.

#### Transparency and openness

We report how we determined sample size, all data exclusions, manipulations, and measures in the study. Raw data and analysis code to replicate analyses are available on the Open Science Framework at the following link: https://osf.io/u4tvc/. Analyses were preregistered on aspredicted.org: https://aspredicted.org/56G_GT1.

## Results

### Resting BP and trait measures of social pain sensitivity

**Figure 1.**
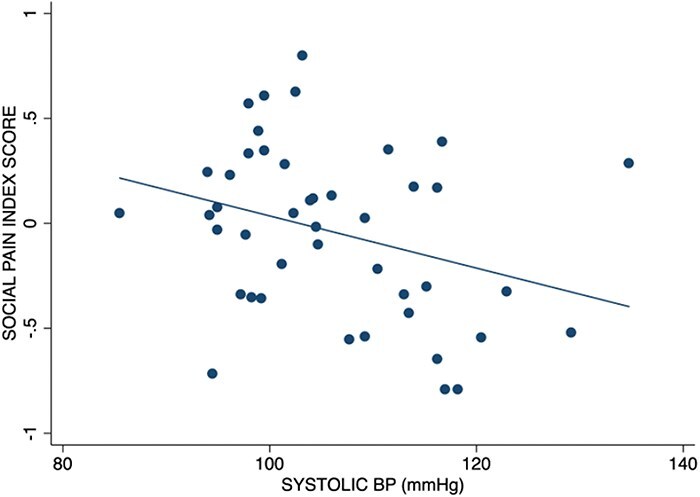
Negative correlation between systolic BP and social pain index score, which is an average of participants’ *z*-scores on three different trait measures of social pain sensitivity (i.e. MSR, BFNE, and HFS). Higher scores (closer to 1) indicate greater sensitivity to social pain.

In replication of previous findings (Inagaki et al. 2018, Umeda et al. 2021, Inagaki and Gianaros 2022), there was a significant association between systolic BP and the social pain sensitivity index score [combined scores from the MSR, BFNE, and HFS, which measure trait sensitivity to social pain; *r *= −0.316, *P *= .018, 90% confidence interval (CI) (−0.526., −0.070.), BF_10_ = 3.044, BF_01_ = 0.329; Fig. 1]. The association remained when controlling for BMI [*t*(43) =  −2.35, *P* = .012, 90% CI (−0.029, −0.005), BF_10_ = 3.222, BF_01_ = 0.310]. There was no association between diastolic BP and the social pain sensitivity index score [*r *= −0.047, *P *= .381, 90% CI (−.295, 0.207), BF_10_ = 0.242, BF_01_ = 4.125].

### Resting BP and reported sensitivity to Cyberball

Participants reported receiving the ball, on average, less than one-third of the time, suggesting they were aware of being excluded from the game (*M* = 15.659%, SD = 9.289%). However, despite previous findings that higher BP was associated with lower sensitivity to Cyberball (Inagaki and Gianaros 2022), this association was not found in the current sample. That is, systolic and diastolic BPs were not related to self-reported sensitivity to Cyberball, measured by the NTS [*r*_SBP_ = −0.096, *P* = .268, 90% CI (−0.339, 0.160), BF_10_ = 0.329, BF_01_ = 3.042; *r*_DBP_ = 0.045, *P* = .385, 90% CI (−.208, 0.293), BF_10_ = 0.152, BF_01_ = 6.589].

### Resting BP and neural activity to Cyberball

Turning to associations between resting BP and neural activity, higher systolic BP was, unexpectedly, associated with higher neural activity in the AI during the exclusion round of Cyberball (compared to the shape-matching control; Table 1). Diastolic BP was likewise associated with both higher AI and dACC activity to exclusion. However, associations did not remain when controlling for BMI [SBP and AI: *t*(42) = 0.45, *P* = .327, 90% CI (−0.013, 0.022); SBP and dACC: *t*(42) = 0.15, *P* = .440, 90% CI (−0.013, 0.016); DBP and AI: *t*(42) = 1.57, *P* = .063, 90% CI (−0.001, 0.034); DBP and dACC: *t*(42) = 1.28, *P* = .105, 90% CI (−0.004, 0.026)].

**Table 1.** Pearson’s correlations and BFs (BF_10_ and BF_01_) for the relationship between BP (systolic and diastolic) and neural activity in AI and dACC to exclusion condition (versus shape-matching and inclusion conditions).

Contrast	BP	Neural region	*r*	90% CI	*P*	BF_10_	BF_01_
Exclusion-shape matching	Systolic	AI	0.276	(**0.024, 0.496**)	.037	0.897	1.115
		dACC	0.134	(−0.124, 0.376)	.195	0.272	3.683
	Diastolic	AI	0.374	(**0.132, 0.573**)	.007	3.617	0.276
		dACC	0.263	(**0.009, 0.485**)	.044	0.771	1.297
Exclusion-inclusion	Systolic	AI	0.161	(−0.097, 0.399)	.151	0.318	3.147
		dACC	0.130	(−0.129, 0.372)	.204	0.265	3.774
	Diastolic	AI	0.282	(**0.030, 0.500**)	.034	0.963	1.039
		dACC	0.273	(**0.020, 0.493**)	.038	0.871	1.148

Notably, these represent the relationships before controlling for BMI and the statistically significant correlations did not hold after controlling for BMI. Statistically significant 90% confidence intervals (CIs) are in bold.

There were no significant associations between systolic BP and AI or dACC activity in the exclusion round compared to the inclusion round (Table 1). However, diastolic BP was associated with AI and dACC activity during the exclusion round of Cyberball compared to the inclusion round. Again, these associations did not hold when controlling for BMI [DBP and AI: *t*(42) = 1.31, *P* = .100, 90% CI (−0.005, 0.041); DBP and dACC: *t*(42) = 1.52, *P* = .069, 90% CI (−0.002, 0.041)].

As a test of whether the subjective experience of Cyberball relates to neural activity to the same experience, associations between sensitivity to Cyberball (as measured via the NTS scale) and brain activity were run, but no associations emerged for the exclusion round compared to the shape-matching or inclusion round (*P* > .43).

### Resting BP and general emotional responding

There were no associations between BP and valence and arousal ratings to the negative or positive images (Table 2), nor between BP and dACC and AI activity to emotional images (Table 3).

**Table 2.** Pearson’s correlations and BFs (BF_10_ and BF_01_) for the relationship between BP (systolic and diastolic) and valence and arousal to negative and positive emotional images.

Rating type	BP	Image type	*r*	90% CI	*P*	BF_10_	BF_01_
		Negative	0.243	(−0.009, 0.466)	.056	0.637	1.571
Valence	Systolic						
		Positive	−0.023	(−0.273, 0.230)	.442	0.190	5.268
		Negative	0.168	(−0.088, 0.402)	.139	0.333	3.004
	Diastolic						
		Positive	−0.195	(−0.425, 0.060)	.103	0.408	2.450
		Negative	0.139	(−0.116, 0.378)	.183	0.279	3.586
Arousal	Systolic						
		Positive	0.023	(−0.229, 0.273)	.440	0.190	5.265
		Negative	−0.133	(−0.372, 0.123)	.195	0.269	3.724
	Diastolic						
		Positive	−0.047	(−.295, 0.207)	.382	0.196	5.095

No significant correlations were found.

**Table 3.** Pearson’s correlations and BFs (BF_10_ and BF_01_) for the relationship between BP (systolic and diastolic) and neural activity in the AI and dACC to negative and positive (versus neutral) image viewing.

Contrast	BP	Neural region	*r*	90% CI	*P*	BF_10_	BF_01_
Negative-neutral	Systolic	AI	−0.091	(−.338, 0.167)	.280	0.224	4.467
		dACC	−0.148	(−.388, 0.110)	.172	0.294	3.407
	Diastolic	AI	−0.033	(−.285, 0.223)	.416	0.194	5.150
		dACC	−0.118	(−.362, 0.140)	.225	0.251	3.991
Positive-neutral	Systolic	AI	0.003	(−.251, 0.258)	.491	0.190	5.261
		dACC	−0.092	(−.338, 0.166)	.279	0.224	4.458
	Diastolic	AI	0.015	(−.240, 0.269)	.461	0.191	5.238
		dACC	−0.011	(−.265, 0.244)	.472	0.190	5.250

No significant correlations were found.

### Neural activity as a mediator

Analyses were run to examine whether neural activity to Cyberball mediates the relationship between BP (both systolic and diastolic) and state and trait measures of sensitivity to social pain (social pain index). However, there were no significant mediations identified (all CIs included 0).

## Discussion

Experiences of social pain are common, but sensitivity to such experiences differ between individuals. The current study tested a potential cardiovascular predictor of individual differences in reported and neural sensitivity to social pain—resting BP—and is the first to examine both a social pain and general emotional task together, within participants. Results replicate previous findings linking BP with trait measures of sensitivity to social pain and also show no reliable associations between BP and neural activity to experiencing exclusion or viewing emotional images.

Our findings are consistent with previous research demonstrating associations between BP and trait sensitivity to social pain (Inagaki et al. 2018, Umeda et al. 2021, Inagaki and Gianaros 2022). Specifically, higher systolic BP is associated with lower scores on a social pain index that combined three different self-report measures of sensitivity to social pain, even after adjusting for BMI, a well-known correlate of resting BP. Replication and extension of this finding to a broader measure of trait sensitivity to social pain suggests the association is not specific to one type of measure of social pain (i.e. not just Mehrabian’s MSR used in our prior studies). BFs likewise suggest the association is consistent.

Although there were associations between BP and trait measures of sensitivity to social pain, there were no associations between BP and state measures of self-reported sensitivity to social pain following an experience of exclusion. This was surprising given previous findings linking BP with responses to the same social pain task, Cyberball (Inagaki and Gianaros 2022). It is difficult to know whether the lack of an association in the current sample was an issue of sample size or whether BP–social algesia findings are more reliable for trait versus state measures of sensitivity to social pain. These possible dissociations merit further study.

Regarding neural findings, there were no reliable associations between BP and AI or dACC activity to exclusion. Although there were significant correlations between systolic BP and neural activity in the AI, as well as between diastolic BP and neural activity to exclusion in the dACC and AI, associations did not remain when controlling for BMI. There are various possible explanations for the lack of reliable associations. First, it may be that there are indeed associations between BP and neural responses to exclusion, but that the current study lacked the power to illuminate this link due to a relatively small sample size. Therefore, further investigation with a larger sample size may allow for a better understanding of true associations between BP and neural activity to exclusion. Additionally, in the current study, we used an ROI approach, which was preregistered. However, it is possible that other analysis methods—such as multivariate pattern, time series, or functional connectivity analysis—may illuminate associations between BP and neural activity in the AI and dACC that are not captured when examining mean activity change in specific regions. For example, multivariate patterns in the same neural regions could differ from mean activity change as examined in the current study. Lastly, it is possible that while the neural correlates of social exclusion may reflect state experiences of social pain, they may not exhibit psychometric properties that are suited for indicating trait-like phenotypes. If this is the case, then these findings may indeed parallel the null associations between state self-reports and BP and statistical associations between trait self-reports and BP. Thus, future studies could incorporate analysis methods beyond examining mean activity in specific ROIs. Another possibility is that there are indeed no relationships between BP and neural activity to exclusion in the dACC and AI. If this is the case, examining this link with a larger sample size would still help to better clarify the associations at play.

We also aimed to tease apart associations between BP and social pain versus general emotional responding, as previous work suggests that higher BP may dampen general emotional responding but does not consider social responding (Pury et al. 2004, McCubbin et al. 2014). One study suggested there is no association between BP and sensitivity to an acute emotional experience in which individuals watched videos of others responding to aversive stimuli (Inagaki and Gianaros 2022). Consistent with this, the current study found no reliable associations between BP and dACC or AI activity to viewing emotional images. Although these findings were in line with the hypothesis that an individual’s BP does not predict their sensitivity to emotional responding, results should be interpreted with caution for the same reasons as mentioned earlier, which include the study’s small sample size and examination of only mean change in neural activity. Nonetheless, BFs suggest that there is indeed no relationship between BP and neural activity to emotional responding. In the future, the current findings should be replicated with a larger sample size. Additionally, studies may test associations between BP and other types of nonsocial emotional tasks, such as those used in previous studies testing the emotional dampening hypothesis (e.g. the Perception of Affect Task), or tasks that elicit stronger emotional responses like fear or anger.

It is also important to mention potential drawbacks associated with task-based imaging and ROI-only approaches. Weaknesses of task-based imaging include a lack of test–retest reliability and internal consistency (Elliott et al. 2020). As for ROI approaches, there are both benefits and limitations. Specifically, ROI approaches can lead to more parsimonious analyses, as fewer statistical tests can be run (Poldrack 2007), but they may also neglect the contribution of other brain regions and thus produce biased results. To overcome the limits of task-based imaging and the ROI-only approach, future studies may instead consider resting state scans during which individuals relive previous experiences, along with the use of the alternative imaging analysis approaches mentioned.

Limitations of the study included a small sample size, alongside the difficulty of linking both physiological and psychological experiences with neural activity. That is, greater power is generally needed to examine relationships between brain mechanisms and physiological or psychological experiences, requiring a larger sample size. This is made difficult by the intensive, resource-heavy nature of running fMRI scans. Nonetheless, in the future, the use of a larger sample or different analysis techniques could promote more robust results. Findings are also correlational. Thus, they do not provide an understanding of the direction of associations between BP and sensitivity to social pain. In the future, examining a causal relationship between BP and sensitivity to social pain may involve the manipulation of BP prior to an experience eliciting social pain or longitudinal studies to clarify the causal direction.

Another limitation is the possible weakness of the Cyberball task as a neural measure of social exclusion. Cyberball is a task that has been widely used to effectively increase social pain in participants ranging from 18 to 86 years old via social rejection (Williams and Sommer 1997, Williams et al. 2000, Hawkley et al. 2011, Löckenhoff et al. 2013). However, other tasks that present multiple trials of social pain may provide a more reliable assessment of sensitivity to social pain, at least at the level of the brain. It is also important to acknowledge that Cyberball represents only one instance of social pain and does not necessarily account for all types of social pain let alone exclusion. Therefore, future work may investigate different paradigms of social pain to increase specificity on possible relationships between BP and sensitivity to social pain. Examples of other social pain paradigms that elicit activity in the AI and dACC include receiving negative feedback from others (Somerville et al. 2006, Eisenberger et al. 2011), reliving a recent romantic breakup (Kross et al. 2011) or other previous experience of social pain (Meyer et al. 2015), and experiencing rejection during a simulated dating experience (Hsu et al. 2020).

In conclusion, the current study contributes further support for a relationship between BP and sensitivity to social pain, although more work is needed to elucidate the mechanisms underlying this link. This includes examining involvement of the AI and dACC—among other neural regions—in BP, social pain sensitivity, and emotional reactivity.

## Supplementary Material

nsaf025_Supp

## Data Availability

Data supporting the findings of this study are available at https://osf.io/u4tvc/.
